# Influence of Mitral Annular Calcification Assessed by Cardiac Computed Tomography on Procedural and Clinical Outcomes of Transcatheter Aortic Valve Implantation

**DOI:** 10.3390/medicina62061206

**Published:** 2026-06-22

**Authors:** Yusuf Ziya Şener, Sadberk Lale Tokgözoğlu, Selin Ardalı Düzgün, Uğur Nadir Karakulak, Ahmet Hakan Ateş, Mehmet Levent Şahiner, Ergün Barış Kaya, Enver Atalar, Necla Özer, Tuncay Hazırolan, Kudret Aytemir

**Affiliations:** 1 Department of Cardiology, Faculty of Medicine, Hacettepe University, Ankara 06230, Türkiye; laletok@gmail.com (S.L.T.); ukarakulak@gmail.com (U.N.K.); ahates06@yandex.com (A.H.A.); mleventsahiner@yahoo.com (M.L.Ş.); doctorkaya@yahoo.com (E.B.K.); eattalar1@gmail.com (E.A.); dr_n.ozer@yahoo.com (N.Ö.); draytemir@yahoo.com (K.A.); 2 Department of Internal Medicine, Yavuzeli District State Hospital, Gaziantep 27970, Türkiye; 3 Department of Radiology, Faculty of Medicine, Hacettepe University, Ankara 06230, Türkiye; selinardali@gmail.com (S.A.D.); tuncayhazirolan@yahoo.com (T.H.)

**Keywords:** aortic stenosis, mitral annular calcification, pacemaker, transcatheter aortic valve implantation, TAVI

## Abstract

*Background and Objectives*: Transcatheter aortic valve implantation (TAVI) is the standard therapy for patients with severe aortic stenosis at intermediate or high surgical risk. Mitral annular calcification (MAC) is frequently observed in this population and has been linked to adverse cardiovascular outcomes. This study evaluated the association between MAC and TAVI-related complications and mortality, and identified predictors of all-cause mortality and permanent pacemaker implantation (PPI) following TAVI. *Materials and Methods*: Patients undergoing self-expanding TAVI between January 2010 and June 2020 were retrospectively analyzed. Outcomes included TAVI-related complications, in-hospital and long-term mortality, and predictors of all-cause mortality and PPI. *Results*: A total of 245 patients (98 men [40%], mean age 76.3 ± 8.3 years) were included. Mean left ventricular ejection fraction was 54.8 ± 11.4%, and aortic valve area was 0.74 ± 0.14 cm^2^. MAC was present in 148 patients (60.4%). Pericardial effusion (26.4% vs. 12.4%, *p* = 0.013) and acute kidney injury (21.6% vs. 7.2%, *p* = 0.005) were significantly more frequent in patients with MAC. PPI was required in 42 patients (17.8%). In-hospital mortality occurred in 14 patients (5.7%), and all-cause mortality was observed in 89 patients (36.3%) during a median follow-up of 23.1 months (IQR, 11.6–44.3). MAC extension into the left ventricular outflow tract was the only independent predictor of PPI (OR: 3.32, *p* = 0.002). Independent predictors of all-cause mortality included use of renin–angiotensin–aldosterone system blockers (HR: 0.54, *p* = 0.012), hemoglobin level (HR: 0.79, *p* = 0.006), severe MAC (HR: 1.94, *p* = 0.024), and post-TAVI atrial fibrillation (HR: 2.39, *p* = 0.002). *Conclusions*: MAC is common in TAVI patients and is associated with increased procedural complications, including higher rates of pericardial effusion and acute kidney injury. Greater MAC severity independently predicts higher all-cause mortality. In addition, MAC extension into the left ventricular outflow tract is an independent predictor of PPI following self-expanding TAVI, emphasizing the importance of comprehensive pre-procedural imaging.

## 1. Introduction

Aortic stenosis (AS) is common in older adults, with a prevalence of 3.4% among individuals aged ≥ 75 years, and is characterized by dyspnea, fatigue, chest pain, and reduced exercise tolerance; if untreated, it may progress to heart failure and death [1]. Although transcatheter aortic valve implantation (TAVI) has become the preferred treatment for patients with severe AS at intermediate or high surgical risk, recent trials have demonstrated comparable long-term outcomes between TAVI and surgical aortic valve replacement (SAVR) in low-risk patients [2]. Despite advances in TAVI techniques and growing operator experience, complications such as paravalvular leak, conduction disturbances requiring permanent pacemaker implantation (PPI), and stroke remain clinically significant [3].

Mitral annular calcification (MAC) is a chronic degenerative condition characterized by progressive fibrosis and calcification of the mitral annulus. It shares common pathophysiological pathways and risk factors with atherosclerosis, including obesity, hypertension, diabetes mellitus, dyslipidemia, and smoking [4]. The prevalence of MAC increases markedly with age, reaching up to 23% in the general population [5]. MAC is highly prevalent among patients with severe AS, affecting more than half of this population, and has been associated with adverse clinical outcomes and reduced survival [6].

Previous studies have demonstrated an association between the presence of MAC and increased risks of all-cause mortality and PPI following TAVI. However, data addressing the impact of MAC severity on other TAVI-related procedural complications remain limited [7,8]. Therefore, the present study aimed to evaluate the influence of MAC on periprocedural outcomes and to assess its association with mortality in patients undergoing TAVI.

## 2. Materials and Methods

### 2.1. Study Design and Participants

This single-center, retrospective study screened all adult patients (aged > 18 years) who underwent transcatheter aortic valve implantation (TAVI) between January 2010 and June 2020. Patients were excluded if they had prior aortic or mitral valve prostheses, unavailable preprocedural cardiac computed tomography images, incomplete outcome data, or aortic stenosis secondary to metabolic disorders. In addition, patients with isolated aortic regurgitation and bicuspid aortic valves were excluded due to the lack of appropriately designed TAVI devices during the study period and the associated increased risk of inadequate valve anchoring, embolization, and paravalvular leak. Demographic characteristics, comorbid conditions, medication use, echocardiographic parameters, procedural details, and follow-up data were retrieved from the institutional electronic medical records.

### 2.2. Assessment of Mitral Annular Calcification

Cardiac computed tomography angiography (CTA) was performed using dual-source CT scanners (Somatom Definition and Somatom Force, Siemens Healthineers, Erlangen, Germany) with either retrospective or prospective electrocardiography (ECG) gating. Tube current and voltage were automatically adjusted using CareDose4D (Siemens Healthineers), with reference settings of 120 kVp and 320 mAs. Collimation was 2 × 64 × 0.6 mm for Somatom Definition and 2 × 192 × 0.6 mm for Somatom Force scanners. Cardiac images were reconstructed with a slice thickness of 0.6–0.75 mm, while thoracoabdominal aorta and peripheral arterial images were reconstructed at 1 mm using a soft-tissue kernel (Bv36). For non-contrast scans performed for calcium scoring, a slice thickness of 3 mm was used. All images were transferred to a dedicated workstation for analysis.

The presence of calcification in the mitral and aortic valves, extension of mitral calcification toward the aortic annulus, and MAC volume were assessed. MAC severity was graded based on circumferential involvement of the mitral annulus: mild (<1/3), moderate (1/3–1/2), and severe (>1/2). Extension of MAC into the left ventricular outflow tract (LVOT) was defined as calcification extending up to 5 mm below the aortic annulus [7].

**Figure 1:**
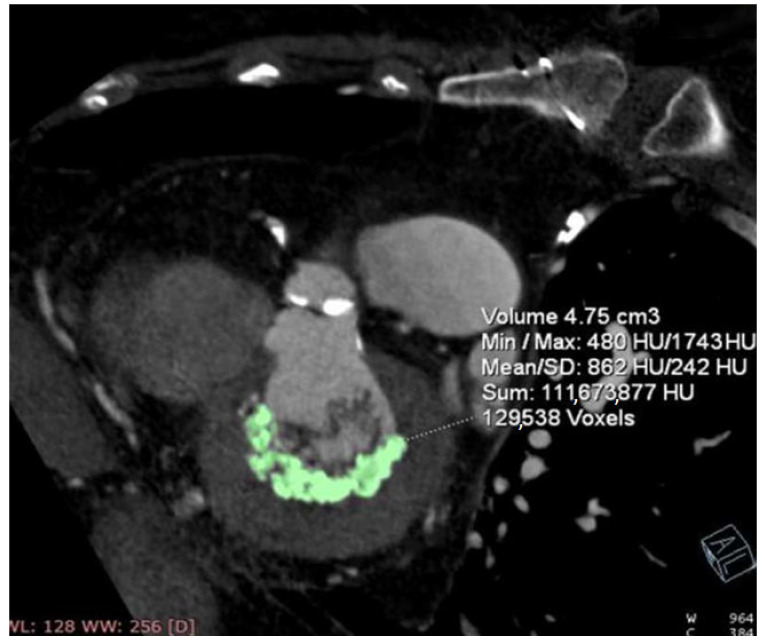
Contrast-enhanced cardiac computed tomography image demonstrating mitral annular calcification. The calcified mitral annulus is highlighted in green using a semiautomated segmentation overlay. Quantitative analysis of the segmented calcification volume and attenuation characteristics is displayed on the image.

All measurements were analyzed using the coronary artery analysis software on a dedicated workstation (Syngo.via VB30, CT Coronary, Siemens Healthineers, Erlangen, Germany). In patients who underwent non-contrast calcium scoring, mitral and aortic valve calcifications were manually segmented using the cardiac CT application, and calcification volume was quantified separately. In patients without calcium scoring scans, such as those with a history of coronary artery bypass grafting, calcification volumes were obtained by manually delineating the calcified regions on consecutive slices and integrating them as a volume of interest. Figure 1 illustrates the assessment of mitral annular calcification volume in a representative patient. All assessments of MAC were performed by an experienced radiologist (S.A.). Intraobserver variability, evaluated using Cohen’s kappa analysis, demonstrated excellent agreement (κ = 0.92, *p* < 0.001).

### 2.3. TAVI Procedure

All procedures were performed under general anesthesia or deep sedation, as determined by the anesthesiology team. Vascular access strategy was guided by preprocedural TAVI-protocol computed tomography, with selection of the most suitable femoral artery for valve delivery. The contralateral femoral artery and vein were cannulated with 6F sheaths, and a temporary transvenous pacing lead was positioned in the right ventricular apex. Peripheral angiography was performed, followed by placement of a pigtail catheter in the non-coronary aortic sinus for hemodynamic assessment. Coronary angiography was performed prior to TAVI when clinically indicated.

Femoral arterial access for valve delivery was achieved either by surgical cutdown in early cases or by percutaneous closure using a Perclose ProGlide™ Suture-Mediated Closure System (Abbott, Santa Clara, CA, USA) in later cases. After crossing the native calcified aortic valve, invasive transvalvular gradients were recorded, and a stiff guidewire was positioned in the left ventricle. The delivery sheath was then upsized to accommodate the transcatheter valve system. Balloon predilatation was selectively performed in patients with severe or asymmetric calcification, small valve area, horizontal aorta, bicuspid anatomy, uncertain valve sizing, or high risk of coronary obstruction. A self-expanding bioprosthetic valve was subsequently deployed under rapid ventricular pacing. Post-deployment aortography was used to assess coronary patency and aortic regurgitation, with balloon postdilatation performed in cases of moderate or severe regurgitation. The procedure was concluded after final angiographic assessment. Echocardiography was performed during the TAVI procedure in cases of suspected cardiac tamponade. Postprocedural echocardiography was routinely performed in all patients immediately after the TAVI procedure, at 24 h, and prior to discharge to assess pericardial effusion as well as cardiac and valvular function.

### 2.4. Clinical Outcomes

The primary endpoint was to assess the impact of MAC on post-TAVI outcomes, including procedural complications (e.g., vascular access complications, pericardial effusion, PPI, and stroke), transcatheter valve performance, and mortality. Vascular access-site complications were defined as the occurrence of hematoma, pseudoaneurysm, or arteriovenous (A–V) fistula confirmed on postprocedural evaluation: these entities are commonly reported as standard access-site injuries in percutaneous cardiovascular procedures [9,10]. Acute kidney injury (AKI) was defined according to the KDIGO criteria as any of the following: an increase in serum creatinine by ≥0.3 mg/dL (≥26.5 µmol/L) within 48 h; an increase in serum creatinine to ≥1.5 times baseline within 7 days; or urine output < 0.5 mL/kg/h for 6 h. AKI was classified as stage 1 if serum creatinine increased 1.5–1.9 times baseline or by ≥0.3 mg/dL, stage 2 if serum creatinine increased 2.0–2.9 times baseline, and stage 3 if serum creatinine increased to ≥4.0 mg/dL or renal replacement therapy was required [11]. Pericardial effusion was defined as the presence of an echo-free space between the visceral and parietal pericardium detected by transthoracic echocardiography in any standard view, in accordance with the American Society of Echocardiography recommendations. Pericardial effusion was classified based on the largest end-diastolic echo-free space as small (<10 mm), moderate (10–20 mm), and large (>20 mm) [12]. Post-TAVI atrial fibrillation (AF) was operationally defined as the occurrence of AF documented after completion of the TAVI procedure, either as a first-time diagnosis in patients without prior history of AF or as a recurrent AF episode in individuals with a history of paroxysmal AF who were in sinus rhythm at the time of the TAVI procedure. All patients underwent routine scheduled ECG at the first follow-up visit, planned approximately one month after TAVI. Routine 24-h Holter monitoring was not performed. The diagnosis of AF was based on surface ECG recordings obtained during follow-up visits or emergency hospital admissions, and on 24-h Holter monitoring in symptomatic patients when clinically indicated.

The secondary endpoint was to identify predictors associated with PPI and mortality following TAVI.

### 2.5. Statistical Analysis

Statistical analyses were performed using Statistical Package for the Social Sciences (SPSS) for Windows version 22.0 (IBM SPSS Inc., Chicago, IL, USA) and R statistical software (version 3.6.3). Data distribution was assessed using histograms and the Kolmogorov–Smirnov test. Normally distributed continuous variables are presented as mean ± standard deviation, whereas non-normally distributed variables are expressed as median (interquartile range [IQR]). Categorical variables are reported as counts and percentages.

Between-group comparisons were conducted using the independent-samples Student’s t test for normally distributed continuous variables and the Mann–Whitney U test for non-normally distributed variables. Categorical variables were compared using the chi-square (χ^2^) test or Fisher’s exact test, as appropriate. A *p* value of less than 0.05 was considered statistically significant. Survival analysis and corresponding curves were generated using the Kaplan–Meier method. Logistic regression analysis was used to identify factors associated with PPI after TAVI. Univariable logistic regression analyses were performed for predefined variables, including sex, age, coronary artery disease, diabetes, beta-blocker use, left ventricular end-diastolic diameter (LVEDD), aortic annulus diameter, aortic valve area, left atrial diameter, presence of MAC, MAC extending into the LVOT, and glomerular filtration rate, to assess their association with PPI. ECG parameters previously reported as predictors of PPI were not included due to unavailable or incomplete data. Factors associated with all-cause mortality were first evaluated using univariable Cox proportional hazards regression; variables with a *p* value < 0.200 were subsequently entered into a multivariable Cox regression model to determine independent predictors of all-cause mortality.

### 2.6. Ethical Approval

The study protocol was approved by the local ethics committee (approval no. 2020/12-34) and registered under study ID GO 20/603.

## 3. Results

### 3.1. Baseline Characteristics

**Figure 2:**
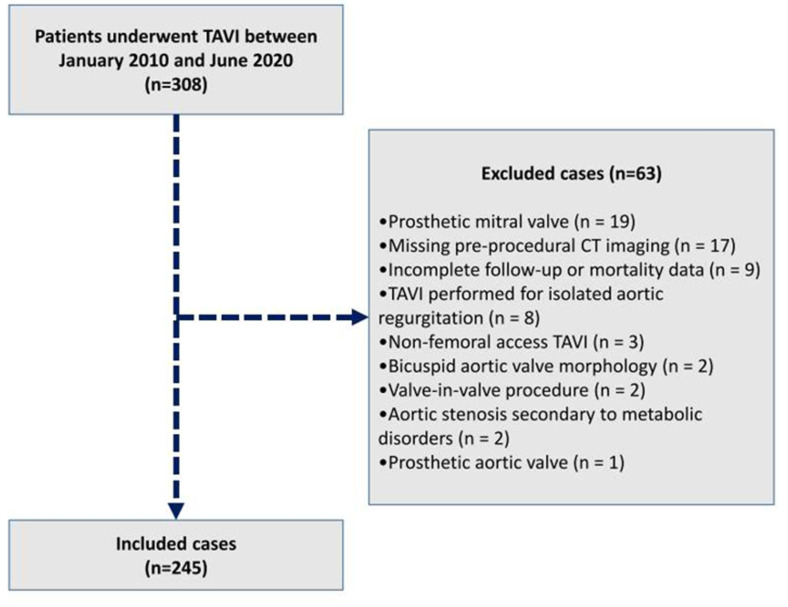
Flowchart of study participant selection.

A total of 245 patients were included (Figure 2). The cohort was predominantly female (60%, *n* = 147), with a mean age of 76.3 ± 8.3 years. Hypertension was present in 186 patients (75.9%), diabetes mellitus in 77 (31.4%), chronic obstructive pulmonary disease in 55 (22.4%), and coronary artery disease in 107 (43.7%). Chronic kidney disease and cerebrovascular disease were each identified in 22 patients (9%). Atrial fibrillation was present in 43 patients (17.6%), and heart failure was observed in 54 patients (22%). At presentation, most patients were classified as NYHA functional class III (*n* = 151). The mean STS score was 9.0 ± 3.4, and the mean logistic EuroSCORE was 32.9 ± 12.8.

Echocardiographic assessment showed a mean left ventricular ejection fraction of 54.8 ± 11.4%. Aortic valve hemodynamics demonstrated a peak gradient of 77.7 ± 21.7 mmHg, a mean gradient of 47.0 ± 14.3 mmHg, and an aortic valve area of 0.74 ± 0.14 cm^2^. MAC was identified in 148 patients (60.4%). The median MAC volume was 0.15 cm^3^ (IQR, 0.0–1.12). Regarding MAC localization, calcification was confined to the anterior annulus in 16 patients (6.5%), the posterior annulus in 70 patients (28.6%), and involved both anterior and posterior annuli in 62 patients (25.3%). MAC severity was graded as mild-to-moderate in 85 patients (34.7%) and severe in 63 patients (25.7%). Extension of mitral annular calcification into the aortic annulus and LVOT was observed in 48 patients (19.6%). Baseline characteristics of the study population and comparisons according to the presence of MAC are summarized in Table 1.

### 3.2. Procedural Characteristics

Regarding procedural characteristics of TAVI, the median procedure time was 60 min (IQR, 50–75), with a median anesthesia duration of 70 min (IQR, 55–87). The mean fluoroscopy time was 11.2 ± 1.1 min. All procedures were performed via the transfemoral approach. The median contrast volume used was 110 mL (IQR, 110–150). Coronary angiography was performed concomitantly with TAVI in 120 patients (49.0%) and in a separate session prior to TAVI in 23 patients (9.4%). Percutaneous coronary intervention was performed concomitantly with TAVI in 49 patients (20.0%) and prior to TAVI in 11 patients. Balloon predilatation was performed in 133 patients (54.3%), and balloon postdilatation in 63 patients (25.7%). The mean balloon diameter was 19.6 ± 1.7 mm for predilatation and 23.7 ± 1.6 mm for postdilatation. The mean prosthetic valve size was 28.4 ± 2.38 mm. A Medtronic CoreValve Evolut-R self-expanding valve was implanted in 200 patients (81.6%), while a St. Jude Medical Portico self-expanding valve was used in 45 patients. ProGlide vascular closure devices were used in 180 patients (73.5%), whereas surgical vascular closure was required in 65 patients (26.5%). Valve-in-valve implantation was necessary in 13 patients (5.3%).

After TAVI, the mean peak transvalvular aortic gradient was 17.9 ± 6.6 mmHg. Aortic regurgitation on aortography was observed in 173 patients (70.6%), including mild regurgitation in 156 patients (63.7%) and moderate regurgitation in 17 patients (6.9%). Valve-in-valve implantation was required in 15 patients (6.1%) overall: this was performed during the index procedure in 13 patients (5.3%), while 2 patients (0.8%) underwent valve-in-valve implantation during follow-up due to valve dysfunction. Procedural characteristics of TAVI are summarized in Table 2.

### 3.3. Mitral Annular Calcification and Periprocedural and Follow-Up Outcomes

Intra-procedural complications were observed in 25 patients (10.2%), including cardiac tamponade (*n* = 8, 3.2%), asystole (*n* = 3, 1.2%), femoral artery dissection (*n* = 8, 3.2%), femoral artery stenosis related to the vascular access closure device (*n* = 3, 1.2%), paroxysmal supraventricular tachycardia (*n* = 1, 0.4%), coronary artery obstruction (*n* = 1, 0.4%), and hypotension due to reduced right coronary artery flow (*n* = 1, 0.4%). Vascular access-site complications occurred in 39 patients (15.9%), including hematoma (*n* = 19, 7.8%), pseudoaneurysm (*n* = 15, 6.1%), arteriovenous fistula (*n* = 2, 0.8%), and wound infection (*n* = 3, 1.2%). Other vascular events comprised aortic rupture in 1 patient (0.4%) and retroperitoneal hematoma in 2 patients (0.8%).

Pericardial effusion developed in 51 patients (20.8%), including 8 cases presenting with cardiac tamponade during the TAVI procedure and the remaining 43 cases occurring during follow-up. Urgent pericardiocentesis was performed in all cases of tamponade developed during the procedure (*n* = 8), and pericardial drainage was hemorrhagic in all patients. Four cases were successfully managed with pigtail catheter insertion, while four required urgent surgical intervention. Among the 43 patients in whom pericardial effusion developed during follow-up, 40 (16.3%) were managed conservatively with medical therapy while pericardiocentesis was required in 3 patients (1.2%). Specifically, percutaneous drainage was sufficient in 2 patients (0.8%), whereas surgical drainage was required in 1 patient (0.4%). Pericardial fluid was seroanguinous in two patients and hemorrhagic in the remaining case.

Acute kidney injury (AKI) occurred in 39 patients (15.9%), with temporary hemodialysis required in 4 (1.6%): no surviving patient required long-term renal replacement therapy. TAVI prosthetic valve endocarditis was documented in 2 patients (0.8%), one of whom underwent surgical aortic valve replacement. Ischemic stroke occurred in 13 patients (5.3%) during follow-up (median 23.1 months; IQR, 11.6–44.3), with 3 events (2.4%) within the first 10 days. Post-TAVI arrhythmic complications occurred in 31 patients (16.2%), including atrial fibrillation in 28 (13.8%) and non-sustained ventricular tachycardia in 3 (2.4%).

Patients with MAC had a higher incidence of pericardial effusion compared with those without MAC (26.4% vs. 12.4%, *p* = 0.013). However, when stratified by severity, the distribution of small, moderate, and large pericardial effusions did not differ significantly between the groups (*p* = 0.890). Acute kidney injury (AKI) was also more frequent in patients with MAC (21.6% vs. 7.2%, *p* = 0.005). In contrast, the distribution of AKI stages (stage 1, 2, and 3) was similar between the groups, with no statistically significant difference (*p* = 0.580). Rates of valve-in-valve implantation, access-site and other vascular complications, post-TAVI aortic regurgitation, infective endocarditis, stroke, and PPI were comparable between the groups. The median follow-up duration was 23.1 months (IQR, 11.6–44.3). Periprocedural and follow-up outcomes according to the presence of MAC are summarized in Table 3.

### 3.4. Mitral Annular Calcification and Permanent Pacemaker Implantation

During post-TAVI follow-up, excluding 9 patients with a pre-existing permanent pacemaker, PPI was required in 42 patients (17.6%). Median time to PPI was 5.5 (IQR, 2–8) days. A multivariable logistic regression model was constructed including five variables, selected based on the total number of events (42 PPI events) to avoid overfitting. Model fit was assessed using the Hosmer–Lemeshow test. Multicollinearity among predictors was evaluated, and overlapping variables were excluded. The final model included coronary artery disease, aortic valve area, left atrial diameter, glomerular filtration rate, and MAC extending into the LVOT. Among these, only MAC extending into the LVOT was independently associated with an increased risk of PPI (OR: 3.32, 95 CI%:1.56–7.05; *p* = 0.002).

### 3.5. Mitral Annular Calcification and Mortality

**Figure 3:**
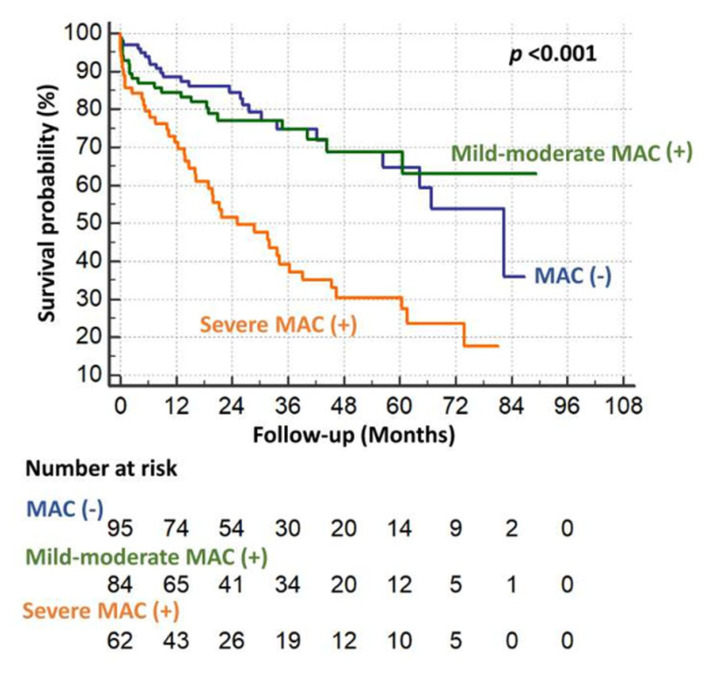
Kaplan–Meier survival curves comparing patients with and without mitral annular calcification.

**Figure 4:**
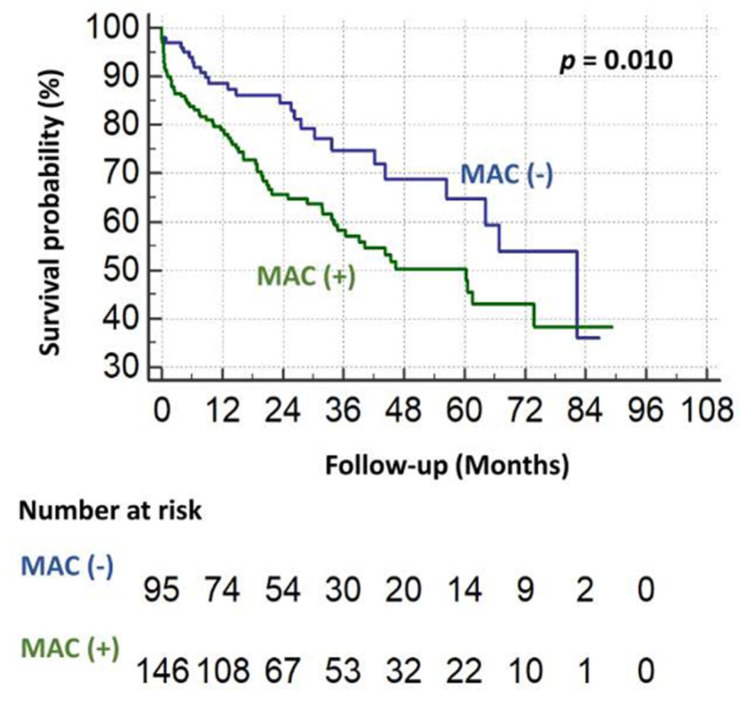
Kaplan–Meier survival curves stratified by severity of mitral annular calcification.

The median follow-up duration was 23.1 months (IQR, 11.6–44.3). During follow-up, 89 patients (36.3%) died, and in-hospital mortality occurred in 14 patients (5.7%). One-, three-, and five-year survival rates were 82%, 64%, and 55%, respectively. All-cause mortality was significantly higher in patients with MAC compared with those without MAC (43.2% vs. 25.8%, *p* = 0.008). Moreover, patients with severe MAC had significantly higher mortality than those with mild-to-moderate MAC (*p* < 0.01) (Figure 3 and Figure 4). The effect of the procedural learning curve on mortality was analyzed by stratifying the cohort into tertiles according to the chronological sequence of TAVI interventions. No significant differences were observed in in-hospital mortality across tertiles (4.8% vs. 6.0% vs. 6.1%; χ^2^ *p* = 0.923), and long-term all-cause mortality was similarly comparable (log-rank *p* = 0.412) (Figure S1).

Factors associated with all-cause mortality during follow-up were evaluated. Variables with a *p* value < 0.200 in univariable Cox regression analysis were included in the multivariable Cox regression model. In multivariable Cox regression analysis, use of renin–angiotensin–aldosterone system (RAAS) inhibitors (HR: 0.54, *p* = 0.012), hemoglobin level (HR: 0.79, *p* = 0.006), development of post-TAVI atrial fibrillation (HR: 2.39, *p* = 0.002), and severe MAC (HR: 1.94, *p* = 0.024) were identified as independent predictors of all-cause mortality (Table 4).

## 4. Discussion

In this study, we demonstrated that mitral annular calcification is highly prevalent among patients with severe aortic stenosis undergoing TAVI, with post-procedural pericardial effusion and AKI occurring more frequently among patients with MAC. We further showed that severe MAC independently predicts all-cause mortality, while MAC extension into the LVOT emerged as an independent predictor of PPI following implantation of self-expanding valves. Beyond confirming previously reported associations with mortality and pacemaker requirement, our findings uniquely highlight the relationship between MAC and specific post-TAVI complications, underscoring the broader clinical impact of MAC in this population.

Previous studies have demonstrated considerable variability in the reported prevalence of MAC among patients with aortic stenosis, with echocardiography-based studies reporting rates ranging from 14.8% to 50.4% [6,13]. In contrast, investigations using cardiac computed tomography (CT) have reported higher prevalence rates, approximately 49–65%, particularly in cohorts with severe aortic stenosis undergoing surgical or transcatheter aortic valve replacement [14,15,16]. When stratified by severity, mild MAC has been reported in about 30% of patients, whereas moderate and severe MAC each occur in approximately 10% [7]. More recently, another study reported a higher prevalence of moderate MAC, with frequencies of 35.4% for mild MAC, 22.0% for moderate MAC, and 7.9% for severe MAC [16]. In the present study, MAC was detected in 60.4% of patients, with mild-to-moderate MAC observed in 34.7% and severe MAC in 25.7%, yielding prevalence and severity rates that are largely consistent with prior CT-based reports. Differences between echocardiographic and CT-based assessments likely reflect the greater sensitivity of CT in identifying and quantifying annular calcification rather than a methodological novelty. Moreover, MAC has been shown to be associated with advanced age, higher body mass index, diabetes mellitus, elevated systolic blood pressure, and increased left atrial volume index [17]. Consequently, the reported prevalence of MAC may vary across studies depending on the distribution and burden of these risk factors within the respective study populations.

Several investigations have examined the association between MAC and clinical outcomes following TAVI. A recent study reported no significant differences in 30-day disabling stroke, bleeding events, vascular complications, or stage 3 acute kidney injury (AKI) between patients with and without CT-diagnosed MAC, provided that moderate or greater mitral regurgitation or mild or greater mitral stenosis was absent [18]. Consistent with these findings, several observational studies have demonstrated comparable in-hospital and 1-year stroke rates regardless of MAC status [7,8,19,20]. A recent systematic review further corroborated these observations, showing no significant difference in stroke incidence between patients with and without MAC at 30 days or 1 year after TAVI [21]. Similarly, major bleeding and vascular access-site complications were generally comparable between MAC and non-MAC cohorts, although patients with severe MAC appeared to have a higher risk of major bleeding events [7,19,21]. Prior studies also reported similar rates of cardiac tamponade and AKI between these groups [7,19].

Our findings align with the existing literature with respect to stroke, bleeding, and vascular access-site complications, as no significant differences were observed between patients with and without MAC. However, we identified a higher incidence of pericardial effusion following TAVI among patients with MAC, a finding that contrasts with prior reports. This difference is likely attributable to variations in endpoint definitions, as previous studies predominantly focused on cardiac tamponade, whereas our analysis captured pericardial effusions across all severities. Extensive annular and adjacent cardiac calcification may create a rigid and anatomically complex procedural environment, increasing technical challenges during valve crossing, positioning, and deployment. Moreover, calcification involving the LVOT and atrioventricular junction may reduce tissue compliance and increase susceptibility to procedural injury. Lastly, chronic systemic inflammation associated with advanced calcific degeneration may further contribute to exaggerated pericardial inflammatory responses following the intervention.

In addition to pericardial complications, MAC was also associated with an increased incidence of AKI in the present cohort. Although AKI occurred more frequently in patients with MAC, the distribution of AKI severity stages (stages 1, 2, and 3) was largely comparable between groups, with similar rates of stage 3 AKI observed in patients with and without MAC (2.7% vs. 2.1%), aligning with previous reports. The observed difference was primarily driven by a higher incidence of stage 1 AKI among patients with MAC (14.1% vs. 4.1%), suggesting that the increased AKI burden in this population may predominantly reflect mild renal injury rather than severe renal dysfunction. Several mechanisms may account for this association. Patients with severe MAC frequently exhibit diffuse systemic atherosclerosis, endothelial dysfunction, chronic inflammation, impaired microvascular perfusion, and extensive vascular calcification, all of which may reduce renal functional reserve and increase susceptibility to renal hypoperfusion during TAVI. In addition, the greater burden of comorbidities and frailty in these patients may contribute to more technically challenging procedures, prolonged procedural duration, increased hemodynamic instability, and greater contrast exposure. Extensive vascular calcification may further impair renal autoregulatory capacity and exacerbate ischemia–reperfusion injury during transient hypotensive episodes, while chronic inflammatory activation and oxidative stress associated with calcific cardiovascular disease may increase vulnerability to contrast-induced nephrotoxicity. These factors may collectively explain the higher incidence of predominantly mild AKI observed in patients with MAC. Discrepancies between our findings and prior studies are likely related to differences in endpoint definitions and the inclusion of the full spectrum of AKI severity in the present analysis.

The incidence of PPI following TAVI varies considerably across studies, ranging from 2.3% to 36.1% depending on valve type and generation, with a median time to implantation of approximately 3–11 days [22,23,24]. In our cohort, the PPI rate was 17.8%, which is consistent with rates reported in the existing literature. Well-established predictors of post-TAVI PPI include pre-existing right bundle branch block, intraprocedural conduction delay reflected by a ΔHV interval > 10 ms, and pre-procedural atrioventricular conduction vulnerability identified by rapid atrial pacing-induced Wenckebach phenomenon [25]. Additional predictors reported in prior studies include baseline right bundle branch block, first-degree atrioventricular block, shorter membranous septum length, LVOT calcification, and implantation of larger (29-mm) transcatheter valves [26].

The relationship between MAC and PPI after TAVI remains controversial. While Mesnier et al. reported a higher incidence of PPI in patients with MAC, several other studies demonstrated comparable PPI rates between patients with and without MAC [7,18,19,20]. In line with these findings, a systematic review showed no significant difference in either 1-month or 1-year PPI rates according to MAC status [21]. Nevertheless, MAC has been identified as a predictor of PPI in selected cohorts, with subsequent studies suggesting that severe MAC or specific MAC localization (particularly involvement of the A3 segment rather than overall MAC burden) may be more relevant for predicting PPI risk [7,8,27]. Importantly, the analysis of PPI predictors in the present study is limited by the unavailability of key established variables, including baseline conduction abnormalities, membranous septum length, and implantation depth. Therefore, the observed association between MAC-LVOT extension and PPI should be interpreted with caution, as residual confounding cannot be excluded. Nevertheless, our findings extend the existing literature by demonstrating that only MAC extending into the LVOT, rather than the mere presence or severity of MAC, was independently associated with an increased risk of PPI, suggesting that the anatomical extent and spatial relationship of MAC to the conduction system may be more clinically meaningful than global MAC burden when assessing post-TAVI conduction risk.

In contemporary cohorts, in-hospital mortality following TAVI ranges from approximately 0.6% to 9%, and all-cause mortality at a mean follow-up of ~1.8 years has been reported at 20.3% [28,29,30]. Previous observational studies have identified multiple predictors of mortality after TAVI, including low serum albumin levels, higher STS score, end-stage renal disease, advanced age, male sex, coronary artery disease, diabetes mellitus, chronic kidney disease stage ≥ 3, liver disease, congestive heart failure, chronic obstructive pulmonary disease, and atrial fibrillation [31,32,33]. The prognostic role of MAC in post-TAVI mortality remains controversial, with some studies reporting no independent association between MAC burden or anatomical distribution and mortality, whereas others have demonstrated that severe MAC independently predicts adverse survival outcomes [7,8].

In our cohort, the observed all-cause mortality rate was higher than that reported in prior studies (36.3%), which may be attributable to a longer follow-up duration and a higher burden of comorbid conditions. Notably, we identified severe MAC as an independent predictor of all-cause mortality, alongside hemoglobin level, de novo atrial fibrillation, and use of RAAS inhibitors, suggesting that both structural cardiac pathology and systemic clinical factors play a critical role in long-term outcomes after TAVI. Our findings suggest that severe MAC represents a marker of advanced calcific–inflammatory disease associated with atherosclerosis, myocardial fibrosis, and reduced ventricular compliance, thereby conferring an increased risk of mortality.

This study has several limitations. First, because all consecutive patients were included from the inception of the TAVI program, the operators’ learning curve may have influenced the incidence of periprocedural complications, particularly in the early cases, although in-hospital and all-cause mortality rates remained comparable across tertiles stratified by chronological sequence. Second, the definition of pericardial effusion in the present study included the full spectrum of echocardiographically detected effusions rather than only clinically significant or Valve Academic Research Consortium (VARC)-defined tamponade events, which may have increased the observed event rate and limits direct comparison with studies using standardized VARC endpoints. Similarly, although AKI was defined according to KDIGO criteria, the higher AKI incidence observed among patients with MAC was primarily driven by stage 1 events, which may be of limited clinical significance. Third, post-TAVI PPI was guided by a strategy based on 48-h rhythm monitoring, and the absence of routine electrophysiological studies may have led to an underestimation of pacemaker requirements, particularly in patients developing late-onset conduction disturbances after discharge. Incomplete medical records limited the availability of detailed conduction-related variables, including baseline electrocardiographic abnormalities, QRS duration, PR interval, bundle branch block patterns, and detailed postprocedural electrocardiographic findings. The absence of these variables precluded comprehensive assessment of conduction disturbances and may have influenced the interpretation of pacemaker implantation outcomes in the present study.

In addition, AF surveillance relied on intermittent electrocardiography and clinically indicated Holter monitoring rather than systematic continuous rhythm monitoring. Consequently, asymptomatic or transient AF episodes may have been underdetected, potentially introducing ascertainment bias and affecting the sensitivity of post-TAVI AF both as a clinical outcome and as a predictor in survival analyses. Nevertheless, the observed association between clinically detected post-TAVI AF and mortality highlights the prognostic relevance of detected events. Furthermore, all implanted valves were self-expanding. Because valve platform type is a major determinant of post-TAVI conduction disturbances and PPI risk, the observed association between MAC-LVOT extension and PPI may not be directly generalizable to patients treated with balloon-expandable or mechanically expanding valve systems. In addition, the exclusion of bicuspid aortic valve anatomy and non-transfemoral access approaches further limits the applicability of our findings to broader TAVI populations. Moreover, established surgical risk scores such as STS and Logistic EuroSCORE II were originally designed to predict short-term periprocedural mortality. Since the primary endpoint of the present study was overall long-term mortality, formal incremental discrimination analyses beyond these scores were not performed. Future prospective studies are needed to determine whether severe MAC provides additional prognostic value for short-term mortality prediction beyond established risk models. Finally, the unavailability of cause-specific mortality data prevented separate analysis of cardiovascular mortality.

## 5. Conclusions

Mitral annular calcification is highly prevalent among patients undergoing TAVI and is associated with an increased risk of procedural complications, especially pericardial effusion and AKI. While overall PPI rates were comparable to those reported in prior studies, extension of MAC into the LVOT emerged as a strong independent predictor of PPI. Moreover, severe MAC independently predicts worse long-term survival. These findings highlight the prognostic and procedural relevance of MAC, underscoring the need for detailed pre-procedural anatomical assessment and risk stratification to optimize outcomes following TAVI.

## Figures and Tables

**Table 1:** Baseline characteristics of the study population.

	All Cases (*n* = 245)	MAC (+) (*n* = 148)	MAC (−) (*n* = 97)	*p*-Value
**Demographics and Surgical Risk**				
Sex, male, *n* (%)	98 (40)	51 (34.5)	47 (48.5)	0.029 *
Age, years	76.3 ± 8.3	77.2 ± 7.70	75.15 ± 9.12	0.060
BMI, kg/m^2^	27.7 ± 4.9	27.9 ± 5.1	27.5 ± 4.7	0.540
NYHA class	3 (3–4)	3 (3–4)	3 (2.5–4)	0.415
STS score	9.0 ± 3.4	9.3 ± 3.6	8.6 ± 2.8	0.102
Logistic EuroSCORE II	32.9 ± 12.8	30.8 (24.0–42.7)	28.8 (21.5–41.6)	0.270
**Comorbidities,** ***n*** **(%)**				
Hypertension	186 (75.9)	115 (77.7)	71 (73.2)	0.420
Diabetes	77 (31.4)	52 (35.1)	25 (25.8)	0.123
COPD	55 (22.4)	35 (23.6)	20 (20.6)	0.578
CAD	107 (43.7)	60 (40.5)	47 (48.5)	0.222
CKD	22 (9.0)	16 (10.8)	6 (6.2)	0.313
Atrial fibrillation	43 (17.6)	25 (16.9)	18 (18.6)	0.738
Ischemic stroke	22 (9.0)	16 (10.8)	6 (6.2)	0.313
Heart failure	54 (22)	30 (20.3)	24 (24.8)	0.409
**Medications,** ***n*** **(%)**				
Beta blockers	161 (65.7)	90 (60.8)	71 (73.2)	0.046 *
Statins	102 (41.6)	58 (39.2)	44 (45.4)	0.338
RAAS inhibitors	126 (51.4)	74 (50)	52 (53.6)	0.581
MRA	23 (9.4)	8 (5.4)	15 (15.5)	0.008 *
Antiplatelet	153 (62.4)	93 (62.8)	60 (61.9)	0.877
Anticoagulants	45 (18.3)	25 (16.9)	20 (20.6)	0.461
**Echocardiographic measurements**				
LVEDD, mm	48.7 ± 6.25	47.7 ± 5.6	50.3 ± 6.7	0.001 *
LVESD, mm	33.4 ± 7.4	32.3 ± 6.0	35.1 ± 8.8	0.008 *
LVEF, %	54.8 ± 11.4	55.7 ± 10.4	53.4 ± 12.7	0.139
LA diameter, mm	42.9 ± 6.0	43.0 ± 5.8	42.7 ± 6.3	0.704
Aortic annulus, mm	21.9 ± 2.3	21.6 ± 2.1	22.3 ± 2.5	0.020 *
Peak AV gradient, mmHg	77.7 ± 21.7	76.5 ± 21.6	79.6 ± 21.7	0.270
Mean AV gradient, mmHg	47.0 ± 14.3	46.3 ± 14.3	48.1 ± 14.3	0.315
AVA, cm^2^	0.74 ± 0.14	0.7 ± 0.1	0.7 ± 0.1	0.538
MR, *n*, % - Mild - Moderate - Severe	98 (40) 88 (35.9) 59 (24.1)	56 (37.8) 56 (37.8) 36 (24.3)	42 (43.3) 32 (33) 23 (23.7)	0.661
TR, *n*, % - Mild - Moderate - Severe	109 (44.5) 86 (35.1) 50 (20.4)	66 (44.6) 47 (31.8) 35 (23.6)	43 (44.3) 39 (40.2) 15 (15.5)	0.211
AR, *n*, % - Mild - Moderate - Severe	126 (51.4) 70 (28.6) 25 (10.2)	76 (51.4) 37 (25) 18 (12.2)	50 (51.5) 33 (34) 7 (7.2)	0.244
sPAP, mmHg	47.7 ± 16.4	49.02 ± 17.92	45.87 ± 13.90	0.125
**Laboratory Parameters**				
Hemoglobin, gr/dL	11.9 ± 1.7	11.6 ± 1.6	12.3 ± 1.8	0.002 *
Creatinine, mg/dL	0.93 (0.73–1.14)	0.93 (0.72–1.18)	0.93 (0.75–1.11)	0.959
GFR, mL/min/1.73 m^2^	69.9 (53.4–83.4)	69.2 (50.1–82.3)	72.9 (57.2–85.2)	0.273
BNP, pg/mL	372 (148–1050)	372 (144.5–1044)	372 (155–10.76)	0.915

Abbreviations: AR, Aortic regurgitation; AV, Aortic valve; AVA, Aortic valve area; BMI, Body mass index; BNP, Brain-type natriuretic peptide; CAD, Coronary artery disease; CKD, Chronic kidney disease; COPD, Chronic obstructive pulmonary disease; GFR, Glomerular filtration rate; LA, Left atrium; LVEF, Left ventricular ejection fraction; LVEDD, Left ventricular end-diastolic diameter; LVESD, Left ventricular end-systolic diameter; MR, Mitral regurgitation; MAC, Mitral annular calcification; MRA, Mineralocorticoid receptor antagonists; NYHA, New York Heart Association; RAAS, Renin–angiotensin–aldosterone system; sPAP, Systolic pulmonary artery pressure; STS, Society of Thoracic Surgeons Score; TR, Tricuspid regurgitation. * *p* values < 0.005 were considered statistically significant.

**Table 2:** Procedural characteristics of the study population, *n* (%).

	All Cases (*n* = 245)	MAC (+) (*n* = 148)	MAC (−) (*n* = 97)	*p*-Value
Balloon predilatation, *n* (%)	133 (54.3)	73 (49.3)	60 (61.9)	0.054
Predilatation balloon diameter, mm	19.6 ± 1.7	19.43 ± 1.50	19.98 ± 1.93	0.070
Balloon postdilatation, *n* (%)	63 (25.7)	39 (26.5)	24 (25)	0.790
Postdilatation balloon diameter, mm	23.7 ± 1.6	23.76 ± 1.72	23.75 ± 1.59	0.965
Valve manufacturer, *n* (%); - St. Jude - Medtronic	45 (18.4) 200 (81.6)	33 (22.3) 115 (77.7)	12 (12.4) 85 (87.6)	0.073
Valve size, mm	28.4 ± 2.38	28.20 ± 2.42	28.78 ± 2.29	0.064
Coronary angiography, *n* (%) - Pre-TAVI - Concomitant with TAVI	120 (49) 23 (9.4)	72(48.6) 14 (9.5)	48 (49.5) 9 (9.3)	0.992
PCI, *n* (%) - Pre-TAVI - Concomitant with TAVI	49 (20) 11 (4.5)	30 (20.3) 7 (4.7)	19 (19.6) 4 (4.1)	0.963
Concomitant ViV implantation, *n* (%)	13 (5.3)	7 (4.7)	6 (6.1)	0.837
Post-TAVI peak AV gradient, mmHg	17.9 ± 6.6	17.9 ± 6.79	18.06 ± 6.3	0.851
Vascular access closure method, *n* (%) - Surgical closure - ProGlide closure device	65 (26.5) 180 (73.5)	28 (28.9) 69 (71.1)	37 (25) 111 (75)	0.503
Anesthesia type, *n* (%) - General anesthesia - Conscious sedation	53 (21.6) 192 (78.4)	33 (22.3) 115 (77.7)	20 (20.6) 77 (79.4)	0.878
Anesthesia duration, min	70 (55–87)	65 (55–88.75)	70 (60–85)	0.539
Procedure duration, min	60 (50–75)	57.50 (45–75)	60 (50–75)	0.932
Fluoroscopy time, min	11.2 ± 1.1	11.2 ± 1.19	11.2 ± 1.16	0.601
Contrast volume, mL	110 (110–150)	110 (110–150)	110 (110–150)	0.445

Abbreviations: MAC, Mitral annular calcification; PCI, Percutaneous coronary intervention; TAVI, Transcatheter aortic valve implantation; ViV, Valve-in-valve.

**Table 3:** Periprocedural and follow-up outcomes, *n* (%).

	All Cases (*n* = 245)	MAC (+) (*n* = 148)	MAC (−) (*n* = 97)	*p*-Value
Intraprocedural complications	25 (10.2)	17 (11.5)	8 (8.2)	0.546
Aortic rupture	1 (0.4)	1 (0.7)	0 (0)	1.000
Retroperitoneal hematoma	2 (0.8)	0 (0)	2 (2.1)	0.156
ViV TAVI - Concomitant - During follow-up	15 (6.1) 13 (5.3) 2 (0.8)	9 (6.1) 7 (4.7) 2 (1.4)	6 (6.2) 6 (6.2) 0 (0)	1.000 0.837 0.520
Aortic regurgitation - Mild - Moderate	156 (63.7) 17 (6.9)	102 (68.9) 9 (6.1)	54 (55.7) 8 (8.2)	0.107
Peak transvalvular gradient, mmHg	17.9 ± 6.6	17.9 ± 6.8	18.1 ± 6.4	0.858
Vascular access-site complications - Hematoma - Pseudoaneurysm - A-V fistula - Infections	39 15.9) 19 (7.8) 15 (6.1) 2 (0.8) 3 (1.2)	21 (14.2) 10 (6.8) 8 (5.4) 2 (1.4) 1 (0.7)	18 (18.6) 9 (9.3) 7 (7.2) 0 (0) 2 (2.1)	0.462
Permanent PM implantation	42 (17.8)	26 (17.8)	16 (17.8)	1.000
Ischemic stroke	13 (5.3)	7 (4.7)	6 (6.2)	0.837
Pericardial effusion, any	51 (20.8)	39 (26.4)	12 (12.4)	0.013 *
Pericardial effusion, severity - Small - Moderate - Large	40 (16.3) 7 (2.8) 4 (1.6)	31 (20.9) 6 (4.0) 3 (2.0)	9 (9.2) 1 (1.0) 1 (1.0)	0.890
Acute kidney injury, any	39 (15.9)	32 (21.6)	7 (7.2)	0.005 *
Acute kidney injury, stage - Stage 1 - Stage 2 - Stage 3	25 (10.2) 8 (3.2) 6 (2.4)	21 (14.1) 7 (4.7) 4 (2.7)	4 (4.1) 1 (1.0) 2 (2.1)	0.580
TAVI endocarditis	2 (0.8)	0 (0)	2 (2.1)	0.156
Post-TAVI AF	28 (13.8)	17 (13.8)	11 (13.9)	1.000
In-hospital mortality	14 (5.7)	11 (7.4)	3 (3.1)	0.173
All-cause mortality	89 (36.3)	64 (43.2)	25 (25.8)	0.008 *
Follow-up, months	23.1 (11.6–44.3)	20.7 (10.1–45.1)	26.1 (12.6–42.8)	0.292

Abbreviations: A-V, Arteriovenous; PM, Pacemaker; TAVI, Transcatheter aortic valve implantation; ViV, Valve-in-valve. * *p* values < 0.005 were considered statistically significant.

**Table 4:** Predictors of all-cause mortality following TAVI.

	Univariable Cox Regression Analysis		Multivariable Cox Regression Analysis	
	HR (95% CI)	*p*-Value	HR (95% CI)	*p*-Value
**Age, years**	1.04 (1.01–1.06)	0.003 *	1.01 (0.99–1.04)	0.220
**Male sex**	0.61 (0.39–0.96)	0.036 *	0.96 (0.58–1.58)	0.873
**CKD**	2.64 (1.51–4.63)	0.001 *	1.90 (0.78–4.60)	0.153
**Atrial fibrillation**	1.77 (1.07–2.93)	0.024 *	2.07 (0.92–4.68)	0.077
**Statin use**	0.73 (0.48–1.13)	0.166	0.64 (0.39–1.04)	0.646
**RAAS inhibitor use**	0.57 (0.37–0.88)	0.012 *	0.54 (0.34–0.87)	0.012 *
**DAPT**	0.60 (0.37–0.98)	0.044 *	1.93 (0.96–3.90)	0.064
**Anticoagulants**	1.46 (1.16–1.84)	0.001 *	1.07 (0.66–1.74)	0.763
**Mean aortic gradient**	0.98 (0.97–1.00)	0.138	0.98 (0.97–1.00)	0.107
**Mitral regurgitation**	1.49 (1.14–1.94)	0.003 *	1.33 (0.95–1.86)	0.089
**Pulmonary artery pressure**	1.01 (1.00–1.02)	0.042 *	0.99 (0.98–1.01)	0.996
**Hemoglobin, g/dL**	0.70 (0.62–0.80)	<0.001 *	0.79 (0.67–0.93)	0.006 *
**Albumin, g/dL**	0.34 (0.22–0.51)	<0.001 *	0.60 (0.34–1.08)	0.090
**GFR, mL/min/1.73 m^2^**	0.98 (0.97–0.99)	<0.001 *	1.00 (0.99–1.01)	0.414
**NYHA class**	1.06 (1.01–1.11)	0.006 *	1.02 (0.96–1.07)	0.452
**Ischemic stroke** **(Pre-TAVI)**	3.32 (1.65–6.68)	0.001 *	1.33 (0.57–3.14)	0.504
**Post-TAVI AF**	4.18 (2.67–6.53)	<0.001 *	2.39 (1.37–4.19)	0.002 *
**Acute kidney injury**	2.22 (1.28–3.85)	0.004 *	2.19 (0.75–6.36)	0.148
**MAC severity**		<0.001 *		0.036 *
**- No (reference)**	-		-	
**- Mild-moderate**	1.04 (0.59–1.86)		1.03 (0.56–1.90)	0.916
**- Severe**	2.98 (1.81–4.92)		1.94 (1.09–3.47)	0.024 *

Abbreviations: CKD, Chronic kidney disease; DAPT, Dual antiplatelet therapy; MAC, Mitral annular calcification; NYHA, New York Heart Association; RAAS, Renin–angiotensin–aldosterone system; TAVI, Transcatheter aortic valve implantation. * *p* values < 0.005 were considered statistically significant.

## Data Availability

The datasets used in this study are not publicly available due to institutional regulations and patient confidentiality considerations. However, they are available from the corresponding author upon reasonable request.

## Supplementary Materials

The following supporting information can be downloaded at: https://www.mdpi.com/article/10.3390/medicina62061206/s1, Figure S1: Kaplan–Meier survival curves demonstrating comparable all-cause mortality across tertiles stratified by chronological sequence of TAVI procedures.

## References

[1] Angioletti C., Moretti G., Manetti S., Pastormerlo L., Vainieri M., Passino C. (2024). The evolution of TAVI performance overtime: An overview of systematic reviews. BMC Cardiovasc. Disord..

[2] Thyregod H.G.H., Jørgensen T.H., Ihlemann N., Steinbrüchel D.A., Nissen H., Kjeldsen B.J., Petursson P., De Backer O., Olsen P.S., Søndergaard L. (2024). Transcatheter or surgical aortic valve implantation: 10-year outcomes of the NOTION trial. Eur. Heart J..

[3] Zou Q., Wei Z., Sun S. (2024). Complications in transcatheter aortic valve replacement: A comprehensive analysis and management strategies. Curr. Probl. Cardiol..

[4] Elmariah S., Budoff M.J., Delaney J.A., Hamirani Y., Eng J., Fuster V., Kronmal R.A., Halperin J.L., O’Brien K.D. (2013). Risk factors associated with the incidence and progression of mitral annulus calcification: The multi-ethnic study of atherosclerosis. Am. Heart J..

[5] Kato N., Guerrero M., Padang R., Amadio J.M., Eleid M.F., Scott C.G., Lee A.T., Pislaru S.V., Nkomo V.T., Pellikka P.A. (2022). Prevalence and Natural History of Mitral Annulus Calcification and Related Valve Dysfunction. Mayo Clin. Proc..

[6] Vanhaecke P., Bohbot Y., Hucleux E., Hasan J., Tribouilloy C. (2025). Mitral Annular Calcification in Severe Aortic Stenosis: Prognostic Value of Calcification Severity and Mitral Valve Dysfunction. Eur. Heart J. Cardiovasc. Imaging.

[7] Abramowitz Y., Kazuno Y., Chakravarty T., Kawamori H., Maeno Y., Anderson D., Allison Z., Mangat G., Cheng W., Gopal A. (2017). Concomitant mitral annular calcification and severe aortic stenosis: Prevalence, characteristics and outcome following transcatheter aortic valve replacement. Eur. Heart J..

[8] Taguchi T., Maeda K., Yanagino Y., Ohmori T., Ohtani A., Kumagai K., Handa K., Inoue K., Yamada S., Misumi Y. (2025). Effect of mitral annular calcification on outcomes following transcatheter aortic valve replacement. Eur. J. Cardio-Thorac. Surg..

[9] Hetrodt J., Engelbertz C., Gebauer K., Stella J., Meyborg M., Freisinger E., Reinecke H., Malyar N. (2021). Access Site Related Vascular Complications following Percutaneous Cardiovascular Procedures. J. Cardiovasc. Dev. Dis..

[10] Halna du Fretay X., Aubry P., Cavillon A., Moisei R. (2020). Vascular access-site infections in percutaneous cardiac interventions: A significant risk? Infection des abords vasculaires et interventions cardiaques percutanées: Un risque significatif?. Ann. Cardiol. Angeiol..

[11] Khwaja A. (2012). KDIGO clinical practice guidelines for acute kidney injury. Nephron Clin. Pract..

[12] Klein A.L., Abbara S., Agler D.A., Appleton C.P., Asher C.R., Hoit B., Hung J., Garcia M.J., Kronzon I., Oh J.K. (2013). American Society of Echocardiography clinical recommendations for multimodality cardiovascular imaging of patients with pericardial disease: Endorsed by the Society for Cardiovascular Magnetic Resonance and Society of Cardiovascular Computed Tomography. J. Am. Soc. Echocardiogr..

[13] Movahed M.R., Saito Y., Ahmadi-Kashani M., Ebrahimi R. (2007). Mitral annulus calcification is associated with valvular and cardiac structural abnormalities. Cardiovasc. Ultrasound.

[14] Ancona M.B., Giannini F., Mangieri A., Regazzoli D., Jabbour R.J., Tanaka A., Testa L., Romano V., Longoni M., Giglio M. (2017). Impact of Mitral Annular Calcium on Outcomes after Transcatheter Aortic Valve Implantation. Am. J. Cardiol..

[15] Takami Y., Tajima K. (2016). Mitral annular calcification in patients undergoing aortic valve replacement for aortic valve stenosis. Heart Vessel..

[16] Pergola V., Cozac D.A., Savo M.T., Mushtaq S., Motta R., Pedrinelli R., Perrone-Filardi P., Sinagra G., De Amicis M., Chiaruttini M.V. (2025). Prognostic significance of severe mitral annular calcification in aortic stenosis: Implications for aortic valve replacement outcome. Int. J. Cardiol. Heart Vasc..

[17] Kim K.A., Jung H.O., Lee S.Y., Ahn Y., Jung M.H., Chung W.B., Lee D.H., Youn H.J., Han D., Chang H.J. (2025). Differences in risk factors associated with the initiation and progression of mitral annular calcification in asymptomatic individuals. Sci. Rep..

[18] Okuno T., Asami M., Khan F., Praz F., Heg D., Lanz J., Kassar M., Khalique O.K., Gräni C., Brugger N. (2020). Does isolated mitral annular calcification in the absence of mitral valve disease affect clinical outcomes after transcatheter aortic valve replacement?. Eur. Heart J. Cardiovasc. Imaging.

[19] Schaefer A., Sarwari H., Schofer N., Schneeberger Y., Westermann D., Schoen G., Blankenberg S., Reichenspurner H., Schäfer U., Conradi L. (2021). TAVI in Patients with Mitral Annular Calcification and/or Mitral Stenosis. Thorac. Cardiovasc. Surg..

[20] Mesnier J., Urena M., Chong-Nguyen C., Fischer Q., Kikoine J., Carrasco J.L., Terzian Z., Brochet E., Iung B., Himbert D. (2021). Impact of Mitral Annular Calcium and Mitral Stenosis on Outcomes After Transcatheter Aortic Valve Implantation. Am. J. Cardiol..

[21] Ahmad S., Yousaf A., Ghumman G.M., Dvalishvili M., Ahsan M.J., Dilibe A., Reis H.L., Qavi A.H., Szerlip M., Goldsweig A.M. (2024). Outcomes of transcatheter aortic valve replacement in patients with mitral annular calcification and concomitant mitral valve dysfunction: A systematic review and meta-analysis. Cardiovasc. Revasc. Med..

[22] Auffret V., Boulmier D., Didier R., Leurent G., Bedossa M., Tomasi J., Cayla G., Benamer H., Beurtheret S., Verhoye J.P. (2024). Clinical effects of permanent pacemaker implantation after transcatheter aortic valve implantation: Insights from the nationwide FRANCE-TAVI registry. Arch. Cardiovasc. Dis..

[23] Ravaux J.M., Di Mauro M., Vernooy K., Van’t Hof A.W., Veenstra L., Kats S., Maessen J.G., Lorusso R. (2021). One-year pacing dependency after pacemaker implantation in patients undergoing transcatheter aortic valve implantation: Systematic review and meta-analysis. JTCVS Open.

[24] van Rosendael P.J., Delgado V., Bax J.J. (2018). Pacemaker implantation rate after transcatheter aortic valve implantation with early and new-generation devices: A systematic review. Eur. Heart J..

[25] Rao K., Chan B., Bhatia K., Saad N., Baer A., Whalley D., Choong C., Hansen P., Bhindi R. (2025). Prospective Observational Study on the Accuracy of Predictors of Permanent Pacemaker Secondary to High-Grade Atrioventricular Conduction Block After TAVI (CONDUCT-TAVI). Circ. Cardiovasc. Interv..

[26] Høydahl M.P., Kjønås D., Rösner A., Trones Antonsen B., Forsdahl S.H., Busund R. (2025). Predictors of permanent pacemaker implantation after transcatheter aortic valve implantation. Scand. Cardiovasc. J..

[27] Boerlage-Van Dijk K., Kooiman K.M., Yong Z.Y., Wiegerinck E.M., Damman P., Bouma B.J., Tijssen J.G., Piek J.J., Knops R.E., Baan J. (2014). Predictors and permanency of cardiac conduction disorders and necessity of pacing after transcatheter aortic valve implantation. Pacing Clin. Electrophysiol..

[28] Lee A.H., Ng A.C.C., Yong A.S.C., Hyun K., Brieger D., Kritharides L., Chow V. (2021). Outcomes of 1,098 Patients Following Transcatheter Aortic Valve Implantation: A Statewide Population-Linkage Cohort Study. Heart Lung Circ..

[29] Rana M., Niethammer M., Sellin C., Dörge H., Eggebrecht H., Schächinger V. (2023). Development of In-Hospital Outcomes in Patients undergoing Transcatheter Aortic Valve Implantation (TAVI) at an Interdisciplinary Heart Center: A Single-Center Experience of 489 Consecutive Cases. Cardiol. Cardiovasc. Med..

[30] Takeji Y., Taniguchi T., Morimoto T., Shirai S., Kitai T., Tabata H., Kitano K., Ohno N., Murai R., Osakada K. (2024). In-hospital outcomes after SAVR or TAVI in patients with severe aortic stenosis. Cardiovasc. Interv. Ther..

[31] Inayat A., Abbas S., Salman F. (2021). Predictors of Mortality in Patients with Transcatheter Aortic Valve Implantation: A National Inpatient Sample Database Analysis. Cureus.

[32] Nishida K., Saji M., Higuchi R., Takamisawa I., Nanasato M., Tamura H., Sato K., Yokoyama H., Doi S., Okazaki S. (2023). Predictors for all-cause mortality in men after transcatheter aortic valve replacement: A report from the LAPLACE-TAVI registry. Int. J. Cardiol. Heart Vasc..

[33] Thogata H., Garikipati S., Reddy S.S., Abhinav Reddy P., Kumar Jella H. (2023). Long-Term Prognosis and Predictors of Mortality in Patients Undergoing Transcatheter Aortic Valve Replacement: A Retrospective Analysis. Cureus.

