# Supplementary material for: Influence of Mitral Annular Calcification Assessed by Cardiac Computed Tomography on Procedural and Clinical Outcomes of Transcatheter Aortic Valve Implantation
Source: Medicina (Kaunas). 2026 Jun 22;62(6):1206. doi: 10.3390/medicina62061206 (PMC13303351; doi:10.3390/medicina62061206)
Supplement: Supplementary file 1 [file medicina-62-01206-s001.zip › medicina-4310040-supplementary.pdf]

Figure Legend

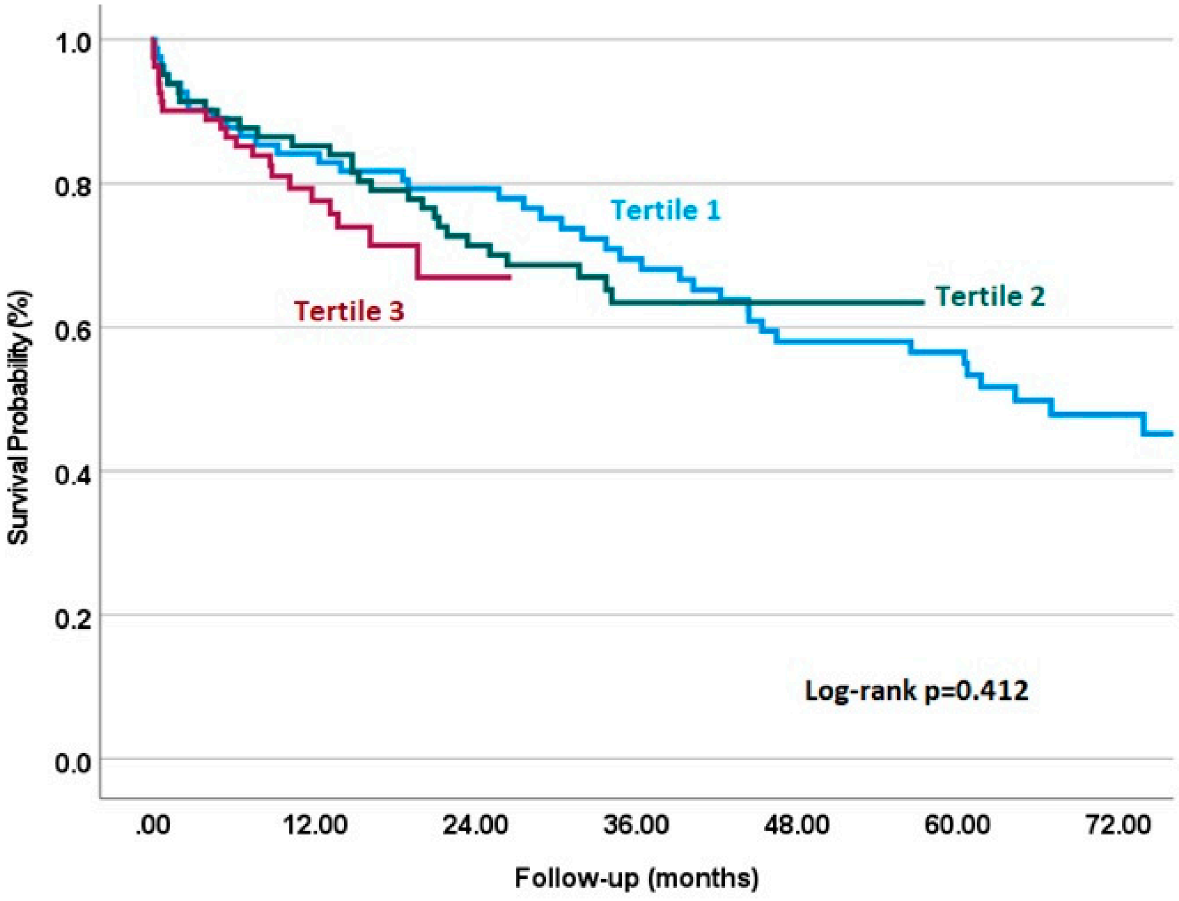

Figure S1. Kaplan–Meier survival curves demonstrating comparable all-cause mortality across tertiles stratified by chronological sequence of TAVI procedures.
